# Search for Potent and Selective Aurora A Inhibitors Based on General Ser/Thr Kinase Pharmacophore Model

**DOI:** 10.3390/ph9020019

**Published:** 2016-04-13

**Authors:** Natalya I. Vasilevich, Victor V. Tatarskiy, Elena A. Aksenova, Denis N. Kazyulkin, Ilya I. Afanasyev

**Affiliations:** ASINEX Company Group, Novie Nauchnie Tekhnologii Ltd., 20 Geroev Panfilovtsev Street, Moscow 125480, Russia; VTatarskiy@asinex.com (V.V.T.); EAksenova@asinex.com (E.A.A.); kazdenis@mail.ru (D.N.K.); iafanasyev@asinex.com (I.I.A.)

**Keywords:** general pharmachophore model, Ser/Thr kinases, Aurora A

## Abstract

Based on the data for compounds known from the literature to be active against various types of Ser/Thr kinases, a general pharmachophore model for these types of kinases was developed. The search for the molecules fitting to this pharmacophore among the ASINEX proprietary library revealed a number of compounds, which were tested and appeared to possess some activity against Ser/Thr kinases such as Aurora A, Aurora B and Haspin. Our work on the optimization of these molecules against Aurora A kinase allowed us to achieve several hits in a 3–5 nM range of activity with rather good selectivity and Absorption, Distribution, Metabolism, and Excretion (ADME) properties, and cytotoxicity against 16 cancer cell lines. Thus, we showed the possibility to fine-tune the general Ser/Thr pharmacophore to design active and selective compounds against desired types of kinases.

## 1. Introduction

Serine/threonine protein kinases are enzymes that phosphorylate the OH group of serine or threonine. Among more than 500 human protein kinases, at least 125 appeared to be serine/threonine kinases (STK) [1]. Inhibitors of Ser/Thr kinases can possess potential therapeutic uses, from treating cancer to immune disorders. Since they were found in a number of mycobacterial organisms, they can be also used for treatment of bacterial infections such as tuberculosis.

A number of attempts have been made to construct a pharmacophore model for various Ser/Thr kinase inhibitors, such as serine/threonine receptor kinase (STPK) inhibitors of tuberculosis, mTor kinase inhibitors, Aurora A and B inhibitors, B-Raf inhibitors, *etc.* [2,3,4,5,6,7,8]. Some of these models are based on the structure-based approach, *i.e.*, docking [3]. Although docking is a promising tool for drug discovery, not all kinases (e.g., tuberculosis PknA) have an X-Ray–resolved structure, and, therefore, preliminary modeling of the binding site is necessary. This results in “double step prediction”: first the modeling of the binding site is done, followed by docking, which in turn decreases the obtained hit rate. Another group of these models has been developed based on the ligand-based approach [4,5,6,7,8]. In general, a more- or less-wide group of molecules is to be aligned and the common structure features are elucidated. Usually, common structure features are developed into more general pharmacophore models [4,5,6,7].

All cited articles are devoted to the search for specific pharmacophore models targeted to a specific group of kinases. We hypothesized that it is possible to find some general features of all serine-threonine inhibitors and to construct a general pharmacophore model. Then this general model might be adjusted to any specific kind of serine-threonine kinase.

## 2. Results and Discussion

To construct the general pharmacophore model, we used known inhibitors of various serine-threonine kinases published in scientific literature and found in the ASINEX proprietary collection. Published STPK inhibitors included inhibitors of tuberculosis kinases, mTor kinase, Aurora A and B kinases, as well as B-Raf kinases [2,3,4,5,6,7,8]. ASINEX proprietary collections included novel compounds tested and found to be active against pknA, pknB kinases. The conformers of all these compounds were generated in MOE (LowModeMD method (software produced by Chemical Computing Group ) with rejection limit of 100, iteration limit of 1000, RMS gradient of 0.005 and MM iteration limit of 500. The RMSD limit was the default and the conformation limit was set to 500). The pharmacophore elucidating module of MOE version 2010.10 was used for further pharmacophore generation with the conformer database obtained (conformations field set to “As-is”). Other settings were the defaults.

**Figure 1:**
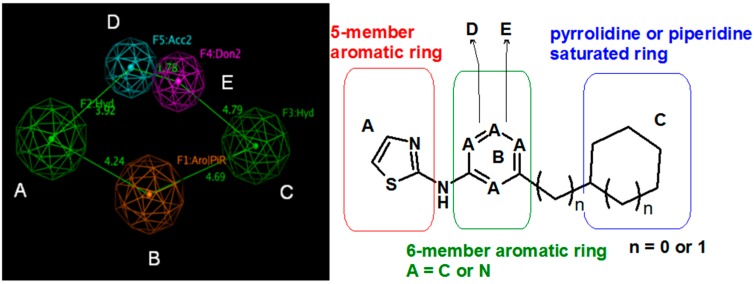
General pharmacophore of serine-threonine kinase inhibitors (MOE 2010.10) and results of its application to ASINEX library.

The resulting general pharmacophore has the shape of a rhomb with a side of around 4–5 A, two hydrophobic groups occupying the opposite nodes, one aromatic group in the third node and H-bond donor and/or acceptor projections located in the remaining corner rather close to each other (Figure 1). The main different feature of this model is that it is more or less planar, unlike the previously developed “volumetric ones” (see. e.g., [1]).

Application of this general pharmacophore model to the ASINEX proprietary library allowed us to find a scaffold fitting to its requirements. This scaffold consisted of a thiazole group connected through an amino group to a nitrogen-containing six-member aromatic cycle, which in turn was bound to a pyrrolidine or piperidine ring directly or via a short linker. In this scaffold, thiazole and saturated cycles served as a hydrophobic group, while the middle-positioned six-member cycle presented a source for H-bond donor or acceptor projections (if one or more “A” moieties in the ring means N).

To verify our hypothesis and the validity of the design, a series of compounds belonging to this scaffold were tested against several Ser/Thr kinases available in-house: Aurora A, Aurora B and Haspin kinases. The results for the most active compounds are shown in Table 1.

To confirm our idea about the possibility of fine-tuning the general pharmacophore for selected types of kinases, we chose Aurora A kinase and tried to adjust the scaffold to its specific requirements. Compound **2**, which showed the best activity against Aurora A (IC_50_ 100 nM), was taken as a starting point and a series of iterations was performed. During the optimization process, three series of compound **2** analogues were synthesized. During the first step we kept the methyl substituent on the thiazole ring and the 2-fluoro-3-chloro-benzoyl substituent on the aliphatic nitrogen, varying both the six-member aromatic ring and an aliphatic *N*-containing cycle tethered to the aromatic one either directly or through a methylene linker. After choosing the best compound in this a series of compounds with methylene-4-piperidine linker, various *N*-acyl and *N*-alkyl substitutions were made. None of these substituents demonstrated superiority compared to the 2-fluoro-3-chloro-benzoyl group. During the last step we varied the thiazole fragment and discovered pyrazole-containing derivatives to be equally or even more active than 4-methyl-thiazole analogues.

As a result we obtained several compounds with excellent potency in the biochemical assay (Table 2).

**Scheme 1:**
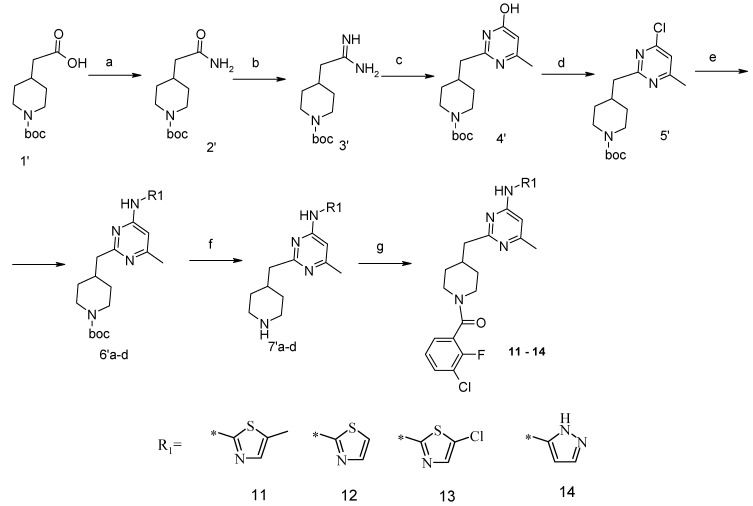
Synthesis of compounds **11**–**14**. *Conditions*: (a) (NH_4_)_2_CO_3_, TBTU, Et_3_N, CH_3_CN, RT, 8 h; (b) triethyloxoniumtetrafluoroborate, CH_2_Cl_2_, RT, 2 h; NH_3_/MeOH, RT, 16 h; (c) CH_3_COCH_2_COOEt, NaO^t^Bu, EtOH, RF, 10 h; (d) POCl_3_, *N*,*N*-dimethylaniline, toluene, RF, 4 h; (e) R_1_NH_2_, Na_2_CO_3_, Pd_2_dba_3_, xantphos, toluene/H_2_O, MW, 140 °C, 2 h; (f) HCl/1,4-dioxane, RT, 4 h; (g) 3-chloro-2-fluoro-benzoic acid, TBTU, Et_3_N, CH_3_CN, RT, 8 h.

Compounds **11**–**14** were synthesized in accordance with Scheme 1. Amide **2′** was prepared from the corresponding Boc-protected amino acid. Compound **1′** was converted into amidine salt **3′** by reaction with triethyloxoniuntetrafluoroborate, followed by treatment with a solution of ammonia in methanol. Cyclization of the amidine obtained with acetoacetic ester in ethanol under reflux gave 6-methylpyrimidone **4′** in 65% yield. This pyrimidones **4′** were treated with three equivalents of phosphorus oxychloride and nine equivalents of dimethylaniline in toluene to give chloride **5′** in 62% yield. A Buchwald reaction with aromatic amines (2-aminothiazoles or 3-aminopyrazoles) led to new derivatives **6′**, and this reaction was performed in a toluene-water mixture with 2.5% mol Pd_2_dba_3_ and 5% mol xantphos and potassium carbonate using a microwave reactor (65%–75% yield). For compound **13** with a 5-chloro-2-thiazole moiety, intermediate **6′** (where R_1_—unsubstituted 2-aminothiazole) was treated with *N*-chlorosuccinimide in dichloroethane at room temperature (yield 70%). To synthesize compound **14** containing a 3-aminopyrazole moiety, the corresponding 3-amino-1H-pyrazole was protected by the tosyl group via reaction with tosyl chloride and sodium hydrocarbonate in acetonitrile. This protective group was easily removed from intermediate **6′** by sodium hydroxide in methanol treatment. After Boc-deprotection, amines **7′** were acylated by 3-chloro-2-fluorobenzoic acid using TBTU as a coupling reagent (yields 85% and more).

**Scheme 2:**
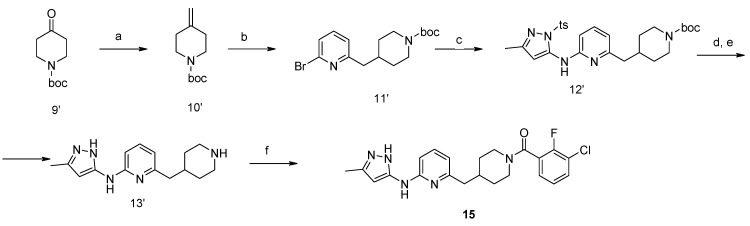
Synthesis of compound **15**. *Conditions*: (a) MePh_3_P^+^I^−^, NaH, THF; (b) 2,6-dibromo-pyridine, 9-BBN, Pd(Ph_3_P)_4_, K_2_CO_3_, THF, RF; (c) 5-methyl-2-(toluene-4-sulfonyl)-2*H*-pyrazol-3-ylamine, Na_2_CO_3_, Pd_2_dba_3_, xantphos, toluene/H_2_O, MW, 140 °C, 2 h; (d) NaOH/MeOH; (e) HCl/1,4-dioxane, RT, 4 h; (f) R_2_COOH, TBTU, Et_3_N, CH_3_CN, RT, 8 h.

Synthesis of compound **15** is shown in Scheme 2. Alkene **10′** was obtained from ketone **9′** via reaction with methyltriphenylphosphonium iodide and sodium hydride in 75% yield. Subsequent treatment with 9-BBN in THF, and 2,6-dibromopyridine in Suzuki reaction conditions gave bromide **11′** in 78% yield. A Buchwald reaction led to intermediate **12′** (70% yield). Compound **15** was prepared by acylation reactions using TBTU as a coupling reagent.

**Scheme 3:**
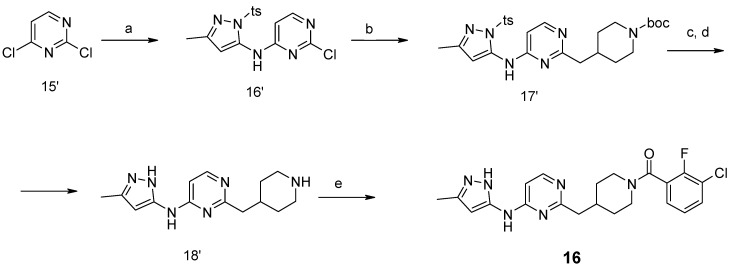
Synthesis of compound **16**. *Conditions*: (**a**) 5-methyl-2-(toluene-4-sulfonyl)-2*H*-pyrazol-3-ylamine, Na_2_CO_3_, Pd_2_dba_3_, xantphos, toluene/H_2_O, MW, 140 °C, 2 hpp; (**b**) 9-BBN, Pd(Ph_3_P)_4_, K_2_CO_3_, THF, RF; (**c**) NaOH/MeOH, RT; (**d**) HCl/1,4-dioxane, RT, 4 h; (**e**) R_2_COOH, TBTU, Et_3_N, CH_3_CN, RT, 8 h.

Compound **16** was synthesized as shown in Scheme 3. A Buchwald reaction with 2,4-dichloropyrimidine **15′** in conditions described above gave a mixture of regioisomers that were separated by column chromatography in 45% yield of target compound **16′**. This compound was involved in a Suzuki reaction with alkene **10′** and 9-BBN, followed by Boc-deprotection, and this led to compound **18′** which was further acylated to compound **16**.

Then three of the most active compounds were tested for selectivity over a number of other kinases and for some of their ADME properties, where they showed rather good results (Table 3).

The five active compounds were screened against 16 cell lines of both tumor and non-tumor origin (Table 4). Overall, Aurora A inhibitors were active against most tumor cell lines with uM IC50, with colon carcinoma Colo320 and acute myeloid leukemia RS4;11 being the most sensitive with submicromolar activity and 10–30 fold selectivity against human fibroblast (HFB) and Mouse Embryonic Fibroblast (MEF) cell lines. Cell lines that were resistant to inhibitors, HL-60, H23 and MDA-MB-231, are also resistant to Aurora A siRNA knockdown [9,10]. Mouse Embryonic Fibroblasts (MEF) with bax^−/−^bak^−/−^ double knockout were less sensitive than with wild-type MEF, indicating that cell death occurred via the mitochondrial apoptotic pathway.

## 3. Experimental Section

### 3.1. Synthesis of Compounds

#### 3.1.1. General Procedure of Buchwald Reaction

A mixture of aryl bromide or aryl chloride (1 mmol), aromatic amine (1 mmol), 9,9-dimethyl-4,5-bis(diphenylphosphino)xantene (0.029 g, 0.05 mmol), *tris*(dibenzylideneacetone)dipalladium(0)-chloroform complex (0.026 g, 0.025 mmol), Na_2_CO_3_ (0.159 g, 1.5 mmol) in toluene (20 mL) and water (1 mL) was stirred at 140 °C under argon atmosphere in microwave reactor for 2 h. The resulting mixture was cooled down, diluted with water and extracted with ethyl acetate. The organic extracts were evaporated to give a crude product, which was further purified by a silica gel column chromatography.

#### 3.1.2. General Procedure of Boc-Deprotection

A solution of Boc-protected compound in 18% HCl in 1,4-dioxane (20 mL) was stirred for 4 h at 25 °C (TLC control). The solvent was removed *in vacuo*, the residue was triturated with ethyl acetate; a precipitate was filtered off, washed with ethyl acetate and dried.

#### 3.1.3. General Procedure of Acylation

First, 3-Chloro-2-fluoro-benzoic acid (63 mg, 0.36 mmol), TBTU (116 mg, 0.36 mmol), amine dihydrochloride (0.3 mmol) and triethylamine (0.168 mL, 1.2 mmol) were dissolved in dry acetonitrile (5 mL). The mixture was stirred for 8 h at room temperature (TLC control). After the reaction was completed the mixture was concentrated to dryness *in vacuo*. Residue was treated with 10% potassium carbonate (10 mL). The resulting mixture was extracted with dichloromethane (2 × 20 mL), organic extract was concentrated and purified by column chromatography.

##### Scheme 1

*4-Carbamoylmethyl-piperidine-1-carboxylic acid tert-butyl Ester* (**2′**). The suspension of 4-carboxymethyl-piperidine-1-carboxylic acid tert-butyl ester (**1′**) (3.04 g, 12.5 mmol), TBTU (4.49 g, 14 mmol), ammonium carbonate (2.4 g, 25 mmol) and triethylamine (5.32 mL, 38 mmol) in dry acetonitrile (80 mL) was stirred for 8 h at room temperature (TLC control). After the reaction was completed the mixture was evaporated to dryness *in vacuo*. Residue was treated with 10% aqueous solution of potassium carbonate (50 mL). The resulting mixture was extracted with dichloromethane (3 × 100 mL), organic extract was evaporated. Purification by column chromatography on silica gel (eluent: hexane/ethyl acetate—1/1) afforded (2′) (2.42 g, 83%). ^1^H-NMR δ_H_ (400 MHz, *d_6_*-DMSO): 0.98 (m, 2H, CH_2_), 1.37 (s, 9H, BOC), 1.61 (m, 2H, CH_2_), 1.79 (m, 1H, CH), 1.96 (m, 2H, CH_2_), 2.70 (m, 2H, CH_2_), 3.87 (d, 2H, CH_2_), 6.61 (br.s., 1H), 7.16 (br.s., 1H). APCI-MS (*m*/*z* (intensity)): 243.2 ([M + H]^+^, 100%).

*4-(4-Hydroxy-6-methyl-pyrimidin-2-ylmethyl)-piperidine-1-carboxylic acid tert-butyl Ester* (**4′**). To a solution of amide (**2′**) (2.42 g, 10 mmol) in dichloromethane (100 mL), triethyloxoniumtetrafluoroborate (1.78 g, 12 mmol) was added at constant stirring; the resulted mixture was stirred for 2 h at room temperature. Then the reaction mixture was concentrated at ~45 °C. The residue was treated with solution of NH_3_/CH_3_OH (pH should be 10), and left to stay overnight. The reaction mixture was evaporated to dryness, and the obtained product (**3′**) was used in the next step without purification. APCI-MS (*m*/*z* (intensity)): 242.1 ([M + H]^+^, 100%).

Crude compound (**3′**) was added to a solution of NaO*^t^*Bu (1.15 g, 12 mmol) in anhydrous ethanol (100 mL). The mixture was stirred at room temperature for 10 min, and then methyl 3-oxobutanoate (1.95 g, 15 mmol) was added. The resulting mixture was refluxed for 10 h (TLC control). Then the reaction mixture was concentrated under reduced pressure, dissolved in water and acidified with 1 N HCl to pH = 5.0 and extracted with ethyl acetate (2 × 100 mL). An organic layer was dried over Na_2_SO_4_ and concentrated in vacuum. The residue was purified by flash-chromatography on silica gel (eluent: hexane/ethyl acetate—1/1). As a result, compound (**4′**) (1.99 g, 65%) was obtained. APCI-MS (*m*/*z* (intensity)): 308.2 ([M + H]^+^, 100%).

*4-(4-Chloro-6-methyl-pyrimidin-2-ylmethyl)-piperidine-1-carboxylic acid tert-butyl Ester* (**5′**). The compound (**4′**) (1.99 g, 6.5 mmol) and dimethylaniline (7.08 g, 58.5 mmol) were dissolved in toluene (100 mL). POCl_3_ (2.99 g, 19.5 mmol) was added dropwise. The reaction mixture was refluxed for 3 h, cooled down to room temperature and poured into water. Organic layer was separated, washed with 1 N HCl and water. Organic layer was concentrated under reduced pressure and purified with flash chromatography (eluent: hexane/ethylacetate 4/1). Yield 1.3 g (62%). ^1^H-NMR δ_H_ (400 MHz, CDCl_3_): 1.20–1.29 (m, 2H, CH_2_), 1.44 (s, 9H, BOC), 1.61 (m, 2H, CH_2_), 2.11 (m, 1H, CH), 2.49 (s, 3H, CH_3_), 2.71 (t, 2H, CH_2_), 2.83 (d, 2H, CH_2_), 4.07 (d, 2H, CH_2_), 7.03 (s, 1H, ArH). APCI-MS (*m*/*z* (intensity)): 325.4 ([M + H]^+^, 100%), 327.4 ([M + H]^+^, 35%).

*4-[4-Methyl-6-(thiazol-2-ylamino)-pyrimidin-2-ylmethyl]-piperidine-1-carboxylic acid tert-butyl Ester* (**6′a**) was prepared using Buchwald reaction, yield 292 mg (75%). ^1^H-NMR δ_H_ (400 MHz, *d_6_*-DMSO): 1.10 (m, 2H, CH_2_), 1.32 (s, 9H, BOC), 1.60 (m, 2H, CH_2_), 2.21 (m, 1H, CH), 2.30 (s, 3H, CH_3_), 2.67 (m, 4H, CH_2_), 3.86 (d, 2H, CH_2_), 6.67 (s, 1H, ArH), 7.12 (m, 1H, ArH), 7.40 (m, 1H, ArH), 11.39 (br.s., 1H, NH). APCI-MS (*m*/*z* (intensity)): 390.12 ([M + H]^+^, 100%).

*4-[4-Methyl-6-(5-methyl-thiazol-2-ylamino)-pyrimidin-2-ylmethyl]-piperidine-1-carboxylic acid tert-butyl Ester* (**6′b**) was prepared using Buchwald reaction, yield 290 mg (72%). ^1^H-NMR δ_H_ (400 MHz, *d_6_*-DMSO): 1.09 (m, 2H, CH_2_), 1.36 (s, 9H, BOC), 1.61 (m, 2H, CH_2_), 2.14 (m, 1H, CH), 2.28 (s, 3H, CH_3_), 2.31 (s, 3H, CH_3_), 2.69(m, 4H, CH_2_), 3.88 (d, 2H, CH_2_), 6.62 (s, 1H, ArH), 7.04 (s, 1H, ArH), 11.13 (s, 1H, NH). APCI-MS (*m*/*z* (intensity)): 404.2 ([M + H]^+^, 100%).

*4-[4-Methyl-6-(5-chloro-thiazol-2-ylamino)-pyrimidin-2-ylmethyl]-piperidine-1-carboxylic acid tert-butyl Ester* (**6′c**) was prepared from (**6′a**) (194 mg, 0.5 mmol) by reaction with *N*-chlorosuccinimide (67 mg, 0.5 mmol) in dichloromethane (20 mL) at room temperature for 4 h. Residue was treated with 10% aqueous solution of potassium carbonate (10 mL). The organic extract was evaporated and purified by column chromatography on silica gel (eluent: hexane/ethyl acetate—4/1) to afford (**6′c**) (148 mg, 70%). ^1^H-NMR δ_H_ (400 MHz, *d_6_*-DMSO): 1.12 (m, 2H, CH_2_), 1.36 (s, 9H, BOC), 1.61 (m, 2H, CH_2_), 2.13 (m, 1H, CH), 2.32 (s, 3H, CH_3_), 2.69 (m, 4H, CH_2_), 3.87 (d, 2H, CH_2_), 6.62 (s, 1H, ArH), 7.41 (s, 1H, ArH), 11.66 (s, 1H, NH). APCI-MS (*m*/*z* (intensity)): 423.4 ([M + H]^+^, 100%), 425.5 ([M + H]^+^, 30%).

*4-[4-Methyl-6-(2H-pyrazol-3-ylamino)-pyrimidin-2-ylmethyl]-piperidine-1-carboxylic acid tert-butyl Ester* (**6′d**). 2-Tosyl-2H-pyrazol-3-ylamine was obtained by reaction between 2H-pyrazol-3-ylamine and tosyl chloride (1 mmol) in presence of sodium bicarbonate (1.2 mmol) in acetonitrile at room temperature for 2 h (yield 60% after recrystallization from ethanol). Buchwald reaction led to tosyl-derivative of (**6′d**), yield 45%. The protective tosyl group was removed by treatment with 0.12 M NaOH in wet methanol at room temperature for 6 h. After reaction completion (TLC control) the mixture was poured into water (20 mL) and extracted with dichloromethane (3 × 20 mL), dried over Na_2_SO_4_ and concentrated in vacuum to give the titled compound (**6′d**). Yield 92%. ^1^H-NMR δ_H_ (400 MHz, *d_6_*-DMSO): 1.04 (m, 2H, CH_2_), 1.35 (s, 9H, BOC), 1.56 (m, 2H, CH_2_), 1.99 (m, 1H, CH), 2.21 (s, 3H, CH_3_), 2.51-2.65 (m, 4H, CH_2_), 3.86 (m, 2H, CH_2_), 6.33 (m, 1H, ArH), 6.85 (m, 1H, ArH), 7.58 (s, 1H, ArH), 9.63 (s, 1H, NH), 12.21 (s, 1H, NH). APCI-MS (*m*/*z* (intensity)): 373.2 ([M + H]^+^, 100%).

*(6-Methyl-2-piperidin-4-ylmethyl-pyrimidin-4-yl)-thiazol-2-yl-amine Dihydrochloride* (**7′a**) was prepared by Boc-deprotection. Yield 287 mg (99%). ^1^H-NMRδ_H_ (400 MHz, *d_6_*-DMSO): 1.57 (m, 2H, CH_2_), 1.86 (m, 2H, CH_2_), 2.33 (m, 1H, CH), 2.56 (s, 3H, CH_3_), 2.84 (m, 2H, CH_2_), 3.07 (m, 2H, CH_2_), 3.19 (m, 2H, CH_2_), 7.03 (br.s, 1H, ArH), 7.41 (m, 1H, ArH), 7.56 (s, 1H, ArH), 8.96 (m, 1H), 9.52 (m, 1H). APCI-MS (*m*/*z* (intensity)): 290.2 ([M + H]^+^, 100%).

*(6-Methyl-2-piperidin-4-ylmethyl-pyrimidin-4-yl)-(5-methyl-thiazol-2-yl)-amine Dihydrochloride* (**7′b**) was prepared by Boc-deprotection. Yield 265 mg (98%). ^1^H-NMR δ_H_ (400 MHz, *d_6_*-DMSO): 1.56 (m, 2H, CH_2_), 1.86 (m, 2H, CH_2_), 2.33 (m, 1H, CH), 2.45 (s, 3H, CH_3_), 2.52 (s, 3H, CH_3_), 2.84 (m, 2H, CH_2_), 2.98 (m, 2H, CH_2_), 3.23 (m, 2H, CH_2_), 6.92 (br.s, 1H, ArH), 7.26 (s, 1H, ArH), 8.95 (m, 1H), 9.20 (m, 1H). APCI-MS (*m*/*z* (intensity)): 304.2 ([M + H]^+^, 100%).

*(5-Chloro-thiazol-2-yl)-(6-methyl-2-piperidin-4-ylmethyl-pyrimidin-4-yl)-amine Dihydrochloride* (**7′c**) was prepared by Boc-deprotection. Yield 136 mg (98%). ^1^H-NMR δ_H_ (400 MHz, *d_6_*-DMSO): 1.55 (m, 2H, CH_2_), 1.84 (m, 2H, CH_2_), 2.28 (m, 1H, CH), 2.51 (s, 3H, CH_3_), 2.83 (m, 2H, CH_2_), 2.98 (m, 2H, CH_2_), 3.23 (m, 2H, CH_2_), 6.92 (s, 1H, ArH), 7.54 (s, 1H, ArH), 8.77 (m, 1H), 9.02 (m, 1H). APCI-MS (*m*/*z* (intensity)): 323.5 ([M + H]^+^, 100%), 325.5 ([M + H]^+^, 32%).

*(6-Methyl-2-piperidin-4-ylmethyl-pyrimidin-4-yl)-(2H-pyrazol-3-yl)-amine Dihydrochloride* (**7′d**) was prepared by Boc-deprotection. Yield 140 mg (99%). APCI-MS (*m*/*z* (intensity)): 273.1 ([M + H]^+^, 100%).

*(3-Chloro-2-fluoro-phenyl)-{4-[4-methyl-6-(thiazol-2-ylamino)-pyrimidin-2-ylmethyl]-piperidin-1-yl}-methanone* (**12**) was prepared by Boc-deprotection. Yield 113 mg (85%). M.p. 207–209 °C. ^1^H-NMR δ_H_ (400 MHz, *d_6_*-DMSO): 1.09–1.26 (m, 2H), 1.64 (m, 1H), 1.80 (m, 1H), 2.23 (s, 3H), 2.35 (m, 1H), 2.65 (m, 2H), 2.84 (m, 1H), 3.04 (m, 1H), 3.33 (m, 1H), 4.43 (m, 1H), 6.45 (s, 1H), 6.89 (d, 1H), 7.28 (m, 3H), 7.62 (m, 2H). ^13^C-NMR δ (400 MHz, *d_6_*-DMSO): 23.45 (CH_3_), 31.30 (CH), 32.06 (CH_2_), 34.43 (CH_2_), 41.31 (CH_2_), 44.82 (CH_2_), 46.59 (CH_2_), 103.22 (CH), 112.25 (CH), 119.9 (d, *J* = 20 Hz, C), 125.80 (d, *J* = 4 Hz, CH), 125.91 (d, *J* = 20 Hz, C), 126.19 (CH), 127.09 (d, *J* = 4 Hz, CH), 131.08 (CH), 137.28 (CH), 152.77 (d, *J* = 247 Hz, C), 157.63 (C), 159.91 (C), 162.40 (C), 164.50 (C), 167.01 (C). APCI-MS (*m*/*z* (intensity)): 445.9 ([M + H]^+^, 100%), 447.7([M + H]^+^, 40%). HRMS (ESI): Calcd for C_21_H_21_ClFN_5_OS ([M + H]^+^), 446.1212; found, 446.1219.

*(3-Chloro-2-fluoro-phenyl)-{4-[4-methyl-6-(5-methyl-thiazol-2-ylamino)-pyrimidin-2-ylmethyl]-piperidin-1-yl}-methanone* (**11**) was prepared by Boc-deprotection. Yield 120 mg (87%). M. p. 211–213 °C. ^1^H-NMR δ_H_ (400 MHz, *d_6_*-DMSO): 1.11–1.27 (m, 2H), 1.62 (m, 1H), 1.78 (m, 1H), 2.27 (s, 3H), 2.34 (s, 3H), 2.72 (m, 2H), 2.83 (m, 1H), 3.05 (m, 1H), 3.34 (m, 1H), 3.45 (m, 1H), 6.61 (s, 1H), 7.05 (s, 1H), 7.25–7.36 (m, 2H), 7.62 (m, 1H), 11.18 (br.s., 1H). ^13^C-NMR δ (400 MHz, *d_6_*-DMSO): 10.87 (CH_3_), 23.46 (CH_3_), 31.25 (CH), 31.98 (CH_2_), 34.43 (CH_2_), 41.29 (CH_2_), 44.71 (CH_2_), 46.56 (CH_2_), 102.41 (CH), 119.9 (d, *J* = 19 Hz, C), 125.48 (C), 125.76 (d, *J* = 4 Hz, CH), 126.09 (d, *J* = 19 Hz, C), 127.08 (d, *J* = 4 Hz, CH), 131.06 (CH), 134.43 (CH), 152.77 (d, *J* = 247 Hz, C), 156.81 (C), 156.91 (C), 162.38 (C), 162.03 (C), 167.16 (C). APCI-MS (*m*/*z* (intensity)): 459.8 ([M + H]^+^, 100%), 461.7 ([M + H]^+^, 37%). HRMS (ESI): Calcd for C_22_H_23_ClFN_5_OS ([M + H]^+^), 460.1369; found, 460.1367.

*(3-Chloro-2-fluoro-phenyl)-{4-[4-(5-chloro-thiazol-2-ylamino)-6-methyl-pyrimidin-2-ylmethyl]-piperidin-1-yl}-methanone* (**13**) was prepared by Boc-deprotection. Yield 130 mg (90%). M. p. 221–223 °C. ^1^H-NMR δ_H_ (400 MHz, *d_6_*-DMSO): 1.12–1.27 (m, 2H), 1.63 (m, 1H), 1.79 (m, 1H), 2.29 (s, 3H), 2.73 (m, 2H), 2.83 (m, 1H), 3.06 (m, 1H), 3.35 (m, 1H), 4.45 (m, 1H), 6.62 (s, 1H), 7.39 (s, 1H), 7.27–7.37 (m, 2H), 7.63 (m, 1H), 11.65 (s, 1H). APCI-MS (*m*/*z* (intensity)): 480.09 ([M + H]^+^, 100%), 482.09 ([M + H]^+^, 68%). HRMS (ESI): Calcd for C_21_H_20_Cl_2_FN_5_OS ([M + H]^+^), 480.0822; found, 480.0825.

*(3-Chloro-2-fluoro-phenyl)-{4-[4-methyl-6-(2H-pyrazol-3-ylamino)-pyrimidin-2-ylmethyl]-piperidin-1-yl}-methanone* (**14**) was prepared by Boc-deprotection. Yield 114 mg (89%). M. p. 125–128 °C. ^1^H-NMR δ_H_ (400 MHz, *d_6_*-DMSO): 1.11–1.34 (m, 2H), 1.62 (m, 1H), 1.83 (m, 1H), 2.16 (m, 1H), 2.25 (s, 3H), 2.60 (m, 2H), 2.81 (m, 1H), 3.06 (m, 1H), 3.29 (m, 1H), 4.43 (m, 1H), 6.35 (s, 1H), 6.89 (s, 1H), 7.26–7.36 (m, 2H), 7.56-7.64 (m, 2H), 9.55 (s, 1H), 12.25 (s, 1H). APCI-MS (*m*/*z* (intensity)): 428.8 ([M + H]^+^, 100%), 430.8 ([M + H]^+^, 41%). HRMS (ESI): Calcd for C_21_H_21_ClFN_6_O ([M + H]^+^), 429.1600; found, 429.1608.

##### Scheme 2

*4-Methylene-piperidine-1-carboxylic acid tert-butyl Ester* (**10′**). NaH in mineral oil (2.28 g, 57 mmol, 60%) was added portion-wise under argon atmosphere to DMSO (35 mL). The reaction mixture was stirred on a water bath up to 75 °C for about 20 min until completion of hydrogen formation. The reaction mixture was cooled down to 20 °C and treated with methyl triphenylsulfonium iodide (23.1 g, 57 mmol). After stirring for about 15 min, ketone (**9′**) (9.96 g, 50 mmol) in THF (15 mL) was added. The mixture was stirred at 25 °C for about 2 h and diluted with water, ethyl acetate and hexane. The organic layer was washed with water (2 × 25 mL), diluted with dichloromethane and washed with brine. The combined aqueous layers were extracted with ethyl acetate (50 mL) and hexane (50 mL). The organic layer was filtered through SiO_2_. The combined filtrate was concentrated and resulted in the title compound. Yield 7.4 g (75%).

*4-(6-Bromo-pyridin-2-ylmethyl)-piperidine-1-carboxylic acid tert-butyl Ester* (**11′**). To a solution of alkene (**10′**) (7.4 g, 37.5 mmol) in 50 mL of anhydrous THF 9-borabicyclo[3.3.1]nonane (90 mL, 0.5 M solution in THF, 45 mmol) was added under argon atmosphere. A resulted solution was stirred at room temperature for 24 h, then 2,6-dibromopyridine (8.9 g, 37.5 mmol), potassium carbonate (7.65 g, 56 mmol), water (15 mL), and catalyst Pd(PPh_3_)_4_ (2.16 g, 1.87 mmol) were added therein. The reaction mixture was refluxed for 4 h, cooled to room temperature, poured into water (100 mL) and extracted with ethyl acetate (2 × 100 mL). The combined organic extracts were evaporated to give a crude oil, which was purified by a silica gel column chromatography to give the titled compound. Yield 10.3 g (78%). ^1^H-NMR δ_H_ (400 MHz, *d_6_*-DMSO): 1.06 (m, 2H, CH_2_), 1.35 (s, 9H, BOC), 1.47 (m, 2H, CH_2_), 1.69–1.87 (m, 2H, CH_2_), 2.65 (m, 3H, CH, CH_2_), 3.84 (d, 2H, CH_2_), 7.26 (m, 1H, ArH), 7.46 (m, 1H, ArH), 7.64 (m, 1H, ArH). APCI-MS (*m*/*z* (intensity)): 355.1 ([M + H]^+^, 100%), 357.0 ([M + H]^+^, 80%).

*4-{6-[5-Methyl-2-(toluene-4-sulfonyl)-2H-pyrazol-3-ylamino]-pyridin-2-ylmethyl}-piperidine-1-carboxylic acid tert-butyl Ester* (**12′**). Compound (**12′**) was prepared by Buchwald reaction, yield 367 mg (70%). 5-Methyl-2-(toluene-4-sulfonyl)-2H-pyrazol-3-ylamine was obtained similar to compound (**6′d**) by reaction of 5-methyl-2H-pyrazol-3-ylamine and tosyl chloride (65%). APCI-MS (*m*/*z* (intensity)): 526.4 ([M + H]^+^, 100%).

*(5-Methyl-2H-pyrazol-3-yl)-(6-piperidin-4-ylmethyl-pyridin-2-yl)-amine Dihydrochloride* (**13′**). Tosyl- and Boc-protective groups of the compound (**12′**) were removed by subsequent treatment with NaOH in methanol and HCl in 1,4-dioxane). Yield 170 mg (90%). ^1^H-NMR δ_H_ (400 MHz, *d_6_*-DMSO): 1.11 (m, 2H, CH_2_), 1.38 (s, 9H, BOC), 1.61 (m, 2H, CH_2_), 1.93 (m, 1H, CH), 2.27 (s, 3H, CH_3_), 2.63–2.79 (m, 4H, CH_2_), 3.78 (d, 2H, CH_2_), 5.96 (s, 1H, ArH), 6.93 (d, 1H, ArH), 7.26 (d, 1H, ArH), 7.94 (m, 2H, ArH, NH), 11.4 (br.s., 1H, NH). APCI-MS (*m*/*z* (intensity)): 372.2 ([M + H]^+^, 100%).

*(3-Chloro-2-fluoro-phenyl)-{4-[6-(5-methyl-2H-pyrazol-3-ylamino)-pyridin-2-ylmethyl]-piperidin-1-yl}-Methanone* (**15**) was prepared by acylation general method. Yield 103 mg (80%). M. p. 120–122 °C. ^1^H-NMR δ_H_ (400 MHz, *d_6_*-DMSO): 1.10–1.25 (m, 2H), 1.58 (m, 1H), 1.74 (m, 1H), 2.02 (m, 1H), 2.18 (s, 3H), 2.53 (m, 2H), 2.79 (m, 1H), 3.00 (m, 1H), 3.32 (m, 1H), 4.43 (m, 1H), 5.90 (s, 1H), 6.50 (d, 1H), 7.05 (s, 1H), 7.23–7.41 (m, 3H), 7.62 (m, 1H), 8.76 (s, 1H), 11.61 (s, 1H). APCI-MS (*m*/*z* (intensity)): 427.8 ([M + H]^+^, 100%), 429.7 ([M + H]^+^, 38%). HRMS (ESI): Calcd for C_22_H_23_ClFN_5_O ([M + H]^+^), 428.1648; found, 428.1642.

##### Scheme 3

*(2-Chloro-pyrimidin-4-yl)-[5-methyl-2-(toluene-4-sulfonyl)-2H-pyrazol-3-yl]-amine* (**16′**). Compound (**16′**) was prepared by Buchwald reaction with 5-methyl-2-(toluene-4-sulfonyl)-2H-pyrazol-3-ylamine. Yield 164 mg (45%). APCI-MS (*m*/*z* (intensity)): 363.6 ([M + H]^+^, 100%), 365.4 ([M + H]^+^, 30%).

*4-{4-[5-Methyl-2-(toluene-4-sulfonyl)-2H-pyrazol-3-ylamino]-pyrimidin-2-ylmethyl}-piperidine-1-carboxylic acid tert-butyl Ester* (**17′**). Compound (**17′**) was prepared by method described for compound (**11′**) from alkene (**10′**) (99 mg, 0.5 mmol) and chloride (**16′**) (164 mg, 0.45 mmol), yield 149 mg (63%). APCI-MS (*m*/*z* (intensity)): 527.4 ([M + H]^+^, 100%).

*(5-Methyl-2H-pyrazol-3-yl)-(2-piperidin-4-ylmethyl-pyrimidin-4-yl)-amine Dihydrochloride* (**18′**). Compound (**18′**) was prepared by method described for compound (**13′**), yield 91 mg (93%). APCI-MS (*m*/*z* (intensity)): 273.1([M + H]^+^, 100%).

*(3-Chloro-2-fluoro-phenyl)-{4-[4-(5-methyl-2H-pyrazol-3-ylamino)-pyrimidin-2-ylmethyl]-piperidin-1-yl}-methanone* (**16**) was prepared by acylation general method. Yield 96 mg (86%). M. p. 115–118 °C. ^1^H-NMR δ_H_ (400 MHz, *d_6_*-DMSO): 1.11–1.25 (m, 2H), 1.60 (m, 1H), 1.76 (m, 1H), 2.17 (s, 3H), 2.62 (m, 2H), 2.81 (m, 1H), 3.04 (m, 1H), 3.33 (m, 1H), 4.45 (m, 1H), 6.08 (s, 1H), 7.01 (s, 1H), 7.25–7.36 (m, 2H), 7.61 (m, 1H), 8.14 (d, 1H), 9.50 (s, 1H), 11.85 (s, 1H). ^13^C-NMR δ (400 MHz, *d_6_*-DMSO): 10.55 (CH_3_), 31.27 (CH), 31.98 (CH_2_), 34.71 (CH_2_), 41.30 (CH_2_), 45.01 (CH_2_), 46.57 (CH_2_), 95.14 (CH), 103.17 (CH), 119.90 (d, *J* = 20 Hz, C), 125.77 (d, *J* = 4 Hz, CH), 126.09 (d, *J* = 20 Hz, C), 127.07 (d, *J* = 4 Hz, CH), 131.06 (CH), 138.28 (CH), 148.28 (C), 152.76 (d, *J* = 247 Hz, C), 155.36 (C), 159.22 (C), 162.36 (C), 167.90 (C). APCI-MS (*m*/*z* (intensity)): 428.8 ([M + H]^+^, 100%), 430.7 ([M + H]^+^, 40%). HRMS (ESI): Calcd for C_21_H_22_ClFN_6_O ([M + H]^+^), 429.1600; found, 429.1602.

### 3.2. Analytical Data for Compounds ***1***–***10*** from the Table 1

*2-Amino-2-methyl-1-(4-(6-(5-propylthiazole-2-ylamino)pyridin-2-yl)piperidin-1-yl)propan-1-one hydrochloride* (**1**). ^1^H-NMR δ_H_ (400 MHz, *d_6_*-DMSO): 0.92 (t, 3H, CH_3_), 1.61 (m, 8H, CH_3_, CH_2_), 1.74 (m, 2H, CH_2_), 1.97 (m, 2H, CH_2_), 2.63 (m, 2H, CH_2_), 3.03 (m, 3H, CH, CH_2_), 6.96 (d, 1H, ArH), 7.03 (d, 1H, ArH), 7.20 (s, 1H, ArH), 8.27 (br.s., 3H, NH, HCl). APCI-MS (*m*/*z* (intensity)): 388.4 ([M + H]^+^, 100%). HRMS (ESI): Calcd for C_20_H_30_N_5_OS^+^ ([M + H]^+^), 388.2166; found, 388.2171.

*(3-Chloro-2-flurophenyl)(3-((4-methylthiazol-2-ylamino)pyrimidin-2-yl)methyl)pirrolidine-1-yl)methanone* (**2**). ^1^H-NMR δ_H_ (400 MHz, *d_6_*-DMSO): 1.67 (m, 1H, CH_2_), 2.02 (m, 1H, CH), 2.32 (s, 3H, CH_3_), 2.82 (m, 2H, CH_2_), 2.90 (m, 1H, CH_2_), 3.22 (m, 1H, CH_2_), 3.35–3.47 (m, 2H, CH_2_), 3.56–3.72 (m, 1H, CH_2_), 6.78 (m, 1H, ArH), 7.07 (m, 1H, ArH), 7.17–7.30 (m, 1H, ArH), 7.37 (m, 1H, ArH), 7.56–7.66 (m, 1H, ArH), 8.28 (m, 1H, ArH), 11.43 (s, 1H, NH). APCI-MS (*m*/*z* (intensity)): 432.7 ([M + H]^+^, 100%). HRMS (ESI): Calcd for C_20_H_30_ClFN_5_OS^+^ ([M + H]^+^), 432.1056; found, 432.1080.

*2-(Dimethylamino)-1-(4-(6-(5-propylthiazol-2-ylamino)pyridin2-yl)piperidin-1-yl)ethanone* (**3**). ^1^H-NMR δ_H_ (400 MHz, *d_6_*-DMSO): 0.91 (t, 3H, CH_3_), 1.65 (m, 2H, CH_2_), 1.72-1.85 (m, 4H, CH_2_), 2.26 (s, 6H, CH_3_), 2.66 (m, 3H, CH_2_), 2.91 (m, 1H, CH), 3.10–3.29 (m, 3H, CH_2_), 4.15 (d, 1H, CH_2_), 4.49 (d, 1H, CH_2_), 6.74 (d, 1H, ArH), 6.83 (d, 1H, ArH), 7.02 (s, 1H, ArH), 10.82 (br.s., 1H, NH). APCI-MS (*m*/*z* (intensity)): 388.4 ([M + H]^+^, 100%). HRMS (ESI): Calcd for C_20_H_30_N_5_OS^+^ ([M + H]^+^), 388.2166; found, 388.2159.

*2-Amino-1-(4-(6-(5-propylthiazol-2-ylamino)pyridin-2-yl)piperidin-1-yl)ethanone* (**4**). ^1^H-NMR δ_H_ (400 MHz, *d_6_*-DMSO): 1.91 (t, 3H, CH_3_), 1.45 (m, 2H, CH_2_), 1.65-1.91 (m, 4H, CH_2_), 2.65 (m, 2H, CH_2_), 2.90 (m, 1H, CH), 3.01–3.19 (m, 4H), 3.36 (m, 2H, CH_2_), 3.89 (m, 1H, CH_2_), 4.47 (m, 1H, CH_2_), 6.64 (d, 1H, ArH), 6.84 (d, 1H, ArH), 7.01 (s, 1H, ArH), 7.55 (m, 1H, ArH). APCI-MS (*m*/*z* (intensity)): 360.4 ([M + H]^+^, 100%). HRMS (ESI): Calcd for C_18_H_26_N_5_OS^+^ ([M + H]^+^), 360.1853; found, 360.1858.

*2-(Methylamino)-1-(4-(6-(5-propylthiazol-2-ylamino)pyridin-2-yl)piperidin-1-yl)ethanone* (**5**). ^1^H-NMR δ_H_ (400 MHz, *d_6_*-DMSO): 1.92 (t, 3H, CH_3_), 1.61 (m, 2H, CH_2_), 1.71–1.90 (m, 4H, CH_2_), 2.31 (m, 3H, CH_3_), 2.64 (m, 3H, CH_2_), 2.91 (m, 1H, CH), 3.02–3.33 (m, 4H, CH_2_), 3.95 (m, 1H, CH_2_), 4.52 (m, 1H, CH_2_), 6.74 (d, 1H, ArH), 6.30 (d, 1H, ArH), 7.01 (s, 1H, ArH), 7.55 (m, 1H, ArH). APCI-MS (*m*/*z* (intensity)): 374.3 ([M + H]^+^, 100%). HRMS (ESI): Calcd for C_19_H_28_N_5_OS^+^ ([M + H]^+^), 374.2009; found, 374.2015.

*(R)-2-Amino-1-(4-(6-(5-propylthiazol-2-ylamino)-pyridin-2-yl)piperidin-1-yl)propan-1-one* (**6**). ^1^H-NMR δ_H_ (400 MHz, *d_6_*-DMSO): 0.91 (t, 3H, CH_3_), 1.11 (m, 3H), 1.57 (m, 3H), 1.67-1.96 (m, 6H), 2.63 (m, 3H), 2.91 (m, 1H, CH), 3.78 (m, 1H, CH_2_), 4.05 (m, 1H, CH_2_), 4.51 (m, 1H, CH_2_), 6.75 (d, 1H, ArH), 6.83 (d, 1H, ArH), 7.02 (s, 1H, ArH), 7.55 (m, 1H, ArH). APCI-MS (*m*/*z* (intensity)): 374.3 ([M + H]^+^, 100%). HRMS (ESI): Calcd for C_19_H_28_N_5_OS^+^ ([M + H]^+^), 374.2009; found, 374.2017.

*2-Amino-1-(4-(4-isopropyl-6-(5-propylthiazol-2-ylamino)pyrimidin-2-yl)piperidin-1-yl)-2-methylpropan-1-one* (**7**). ^1^H-NMR δ_H_ (400 MHz, *d_6_*-DMSO): 1.91 (t, 3H, CH_3_), 1.17 (d, 6H, CH_3_), 1.29 (s, 6H, CH_3_), 1.59 (q, 2H, CH_2_), 1.79–1.93 (m, 4H, CH_2_), 1.65 (m, 2H, CH_2_), 1.80 (m, 1H, CH), 2.92 (m, 3H, CH, CH_2_), 4.75 (m, 2H, CH_2_), 6.63 (s, 1H, ArH), 7.09 (s, 1H, ArH). APCI-MS (*m*/*z* (intensity)): 431.4 ([M + H]^+^, 100%). HRMS (ESI): Calcd for C_22_H_35_N_6_OS^+^ ([M + H]^+^), 431.2588; found, 431.2591.

*2-Amino-1-(4-(4-ethyl-6-(5-propylthiazol-2-ylamino)pyrimidin-2-yl)piperidin-1-yl)-2-methylpropan-1-one* (**8**). ^1^H-NMR δ_H_ (400 MHz, *d_6_*-DMSO): 0.93 (t, 3H, CH_3_), 1.14 (t, 3H, CH_3_), 1.31 (s, 6H, CH_3_), 1.57 (q, 2H, CH_2_), 1.81 (m, 2H, CH_2_), 1.90 (m, 2H, CH_2_), 2.57 (q, 2H, CH_2_), 1.64 (m, 2H, CH_2_), 2.93 (m, 3H, CH, CH_2_), 4.75 (m, 2H, CH_2_), 6.63 (s, 1H, ArH), 7.09 (s, 1H, ArH). APCI-MS (*m*/*z* (intensity)): 417.4 ([M + H]^+^, 100%). HRMS (ESI): Calcd for C_21_H_33_N_6_OS^+^ ([M + H]^+^), 417.2431; found, 417.2344.

*2-Amino-2-methyl-1-(4-(6-(5-phenylthiazol-2-ylamino)pyridine-2-yl)propan-1-one hydrochloride* (**9**). ^1^H NMR δ_H_ (400 MHz, *d_6_*-DMSO): 1.59 (s, 6H, CH_3_), 1.81 (m, 2H, CH_2_), 2.01 (m, 3H), 3.04 (m, 4H), 6.87 (d, 1H, ArH), 6.95 (d, 1H, ArH), 7.27 (m, 1H, ArH), 7.40 (t, 2H, ArH), 7.53 (m, 2H, ArH), 7.66 (t, 1H, ArH), 7.78 (s, 1H, ArH), 8.86 (m, 3H, NH, HCl). APCI-MS (*m*/*z* (intensity)): 422.4 ([M + H]^+^, 100%). HRMS (ESI): Calcd for C_23_H_28_N_5_OS^+^ ([M + H]^+^), 422.2009; found, 422.2014.

*Morpholin-2-yl(4-(6-(5-phenylthiazol-2-ylamino)pyridin-2-yl)piperidin-1-yl)methanone* (**10**). ^1^H-NMR δ_H_ (400 MHz, *d_6_*-DMSO): 1.61–1.92 (m, 5H, CH_2_), 2.63–3.11 (m, 7H, CH, CH_2_), 3.51 (m, 1H, CH), 3.69 (m, 1H, CH_2_), 4.13 (m, 2H, CH_2_), 4.48 (m, 1H, NH), 6.82 (d, 1H, ArH), 6.89 (d, 1H, ArH), 7.25 (m, 1H, ArH), 7.38 (t, 2H, ArH), 7.52 (m, 2H, ArH), 7.61 (t, 1H, ArH), 7.74 (s, 1H, ArH), 11.20 (br.s., 1H, NH). APCI-MS (*m*/*z* (intensity)): 450.23 ([M + H]^+^, 100%). HRMS (ESI): Calcd for C_24_H_28_N_5_O_2_S^+^ ([M + H]^+^), 450.1958; found, 450.1960.

### 3.3. Cytotoxicity Assay

#### 3.3.1. Cell Lines

All cells lines were purchased in ATCC (Manassas, VA, USA), except for human primary fibroblasts (HFB), which were a generous gift from Dashinimaev E.B. (Koltsov Institute of Developmental Biology). H1299 (lung adenocarcinoma), H23 (lung adenocarcinoma), Colo320 (colon carcinoma), RS4;11 (acute lymphoblastic leukemia), PC3 (prostate adenocarcinoma) and HL-60 (acute promyelocytic leukemia) were cultivated in RPMI-1640 medium, with addition of 10% fetal calf serum (HyClone, South Logan, Utah, USA) and 2 mM l-Glutamine at 37 °C, 5% CО_2_. MDA-MB-453 (mammary adenocarcinoma), A549 (lung adenocarcinoma), HCT-116 (colon carcinoma), A431 (epidermoid carcinoma), MDA-MB-231 (mammary adenocarcinoma), BxPC3 (pancreatic adenocarcinoma), MEF (mouse embryonic fibroblasts, SV-40 immortalized), MEF Bax^−/−^/Bak^−/−^ knockout cells (mouse embryonic fibroblasts, SV-40 immortalized, resistant to apoptosis) were cultivated in DMEM medium, with addition of 10% fetal calf serum (HyClone) and 2 mM l-Glutamine at 37 °C, 5% CО_2_.

#### 3.3.2. Cytotoxicity

Cells were seeded in 96-well plates (Corning, NY, USA): 5000 cells per well for adhesive cultures and cells, 25,000 cells per well for suspension cultures. Analyzed substances were added in 0.1–50 μM concentration range, with 2 × dilutions, and incubated for 72 h at 37 °C, 5% CО_2_. After incubation 20 uL of 5 mg/mL of water solution of 3-(4,5-dimethylthiazol-2-yl)-2,5-diphenyltetrazolium bromide (MTT, PANEKO, Moscow, Russia) was added to the cells and they were incubated for additional 4 h. After the incubation the medium was aspirated, cells were dissolved in 100 uL of DMSO and the optical density was measured at 570 nm with a МultiscanFC plate reader (ThermoScientific, Cambridge, MA, USA). The percentage of surviving cells at each dose was calculated by dividing the optical density of treated cells by the optical density of untreated control (which was taken as 100%). The IC50s were calculated in GraphPad Prism 5.0.

## 4. Conclusions

Based on the literature and proprietary data on various Ser/Thr kinase inhibitors, a general pharmachophore model was developed. A search for the molecules fitting to this pharmacophore among the ASINEX library revealed a number of compounds, which were tested and appeared to possess some activity against Aurora A, Aurora B and HaspinSer/Thr kinases.

During optimization of these molecules for Aurora A we found several hits with 3–5 nM activity, which were cytotoxic against 16 cancer cell lines with 10–30 fold selectivity against primary cells.

Thus, we showed the possibility of fine-tuning the general Ser/Thr pharmacophore designed for desired types of kinase to obtain active and selective compounds.

## Figures and Tables

**Table 1:** Activity of compounds fitting to general pharmacophore model against selected Ser/Thr kinases.

No.	Structure	Aurora A, IC_50_, μM	Aurora B, IC_50_, μM	Haspin, IC_50_, μM
**1**	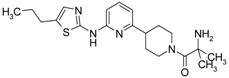	2.06 ± 0.15	1.45 ± 0.17	1.49 ± 0.15
**2**	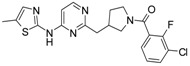	0.10 ± 0.005	3.46 ± 0.36	105.2 ± 11.2
**3**	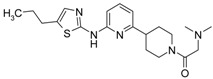	1.95 ± 0.22	1.76 ± 0.16	7.06 ± 0.68
**4**	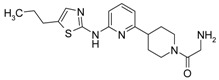	2.08 ± 0.25	1.72 ± 0.19	0.08 ± 0.008
**5**	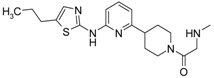	1.44 ± 0.13	0.91 ± 0.08	0.43 ± 0.034
**6**	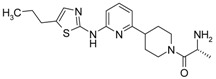	1.67 ± 0.17	1.75 ± 0.18	1.12 ± 0.11
**7**	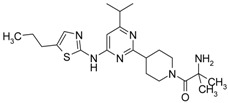	2.79 ± 0.30	2.25 ± 0.23	3.67 ± 0.37
**8**	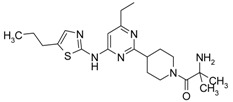	3.58 ± 0.43	2.05 ± 0.24	2.90 ± 0.43
**9**	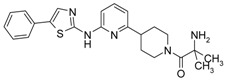	3.17 ± 0.33	8.02 ± 0.80	0.27 ± 0.019
**10**	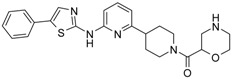	8.38 ± 0.84	0.84 ± 0.09	7.80 ± 0.81

**Table 2:** Compounds optimized for Aurora A kinase inhibition.

No.	Structure	IC50, nM (Mean ± SEM, four Repeats)
**2**	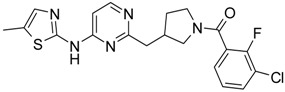	96.0 ± 9.1
**11**	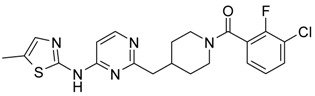	12.0 ± 1.1
**12**	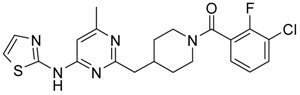	35.1 ± 0.36
**13**	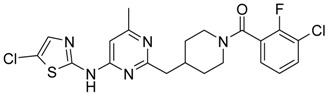	4.1 ± 0.35
**14**	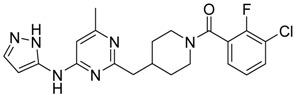	5.3 ± 0.46
**15**	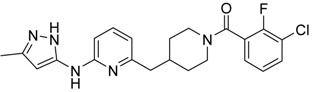	17.5 ± 2.1
**16**	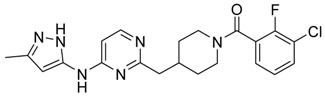	3.5 ± 0.29

**Table 3:** Selectivity and some ADME properties for compounds **13**, **14** and **16**.

Ser/Thr Kinases and ADME ^1^ Parameters	13	14	16
	IC_50_, nM (Mean ± SEM ^2^)	IC_50_, nM (Mean ± SEM)	IC_50_, nM (Mean ± SEM)
Aurora A	4.1 ± 0.35	5.3 ± 0.46	3.5 ± 0.29
Aurora B	232.2 ± 24.1	18,300.0 ± 185.0	4182.5 ± 40.1
c-Kit	17,000.00 ± 165	21,000 ± 250	N/A
EGFR	N/A ^3^	N/A	N/A
CSK	9500.0 ± 101.0	N/A	N/A
EPHA2	9000.0 ± 985.0	N/A	N/A
GSK-3	N/A	109.0 ± 12.1	292.8 ± 31.2
Haspin	N/A	N/A	N/A
JNK3	85.18 ± 9.1	1634.0 ± 167	572.5 ± 56.1
LYN	3270.0 ± 324.0	N/A	N/A
PIM1	N/A	N/A	N/A
PLK1	N/A	N/A	N/A
RON	970.0 ± 101.0	N/A	N/A
CYP1A2 ^4^	N/A	N/A	N/A
CYP2C19	N/A	9597.0 ± 960.0	965.7 ± 94.1
CYP 2C9	N/A	3900.0 ± 401	326.6 ± 33.0
CYP3A4	N/A	15070.0 ± 151	596.2 ± 60.1
HERG	N/A		
Solubility (nephelometry)	<10 μg/mL	>100 μg/mL	>100 μg/mL

^1^ Absorption, distribution, metabolism and excretion; ^2^ Standard error of mean; ^3^ Not available; ^4^ cytochrome P450 isoforms.

**Table 4:** Cytotoxicity of selected Aurora A inhibitors, μМ.

Cell Line	11	12	13	15	16
**HL-60**	5.4 ± 0.3	39.8 ± 3.4	20.9 ± 2.1	38.8 ± 3.2	>50.0
**H23**	3.9 ± 0.15	6.8 ± 0.5	1.2 ± 0.09	>50.0	>50.0
**H1299**	1.7 ± 0.2	5.0 ± 0.4	1.1 ± 0.1	3.7 ± 0.3	1.5 ± 0.12
**PC3**	3.2 ± 0.2	6.0 ± 0.7	3.9 ± 0.4	9.7 ± 1.0	8.3 ± 0.8
**MDA-MB-453**	10.0 ± 1.2	16.3 ± 1.7	8.0 ± 0.79	5.7 ± 0.6	12.0 ± 1.2
**RS4;11**	0.5 ± 0.04	1.5 ± 0.2	1.1 ± 0.11	0.1 ± 0.02	0.3 ± 0.02
**A-549**	5.6 ± 0.5	10.0 ± 1.2	5.1 ± 0.4	5.7 ± 0.6	8.8 ± 0.9
**HCT-116**	3.0 ± 0.4	6.5 ± 0.7	4.4 ± 0.5	2.8 ± 0.3	4.2 ± 0.4
**HFB**	3.6 ± 0.4	3.8 ± 0.4	2.8 ± 0.3	3.0 ± 0.2	3.3 ± 0.3
**A431**	4.6 ± 0.5	10.2 ± 1.2	6.1 ± 0.7	6.8 ± 0.7	9.0 ± 1.0
**Colo-320**	0.8 ± 0.09	2.0 ± 0.15	1.0 ± 0.11	1.0 ± 0.12	1.2 ± 0.03
**MDA-MB231**	7.3 ± 0.8	28.2 ± 0.3	10.5 ± 1.1	>50	>50
**BxPC3**	2.0 ± 0.021	6.0 ± 0.59	2.4 ± 0.14	7.2 ± 0.6	8.1 ± 0.8
**MEFs Dko 48 h**	3.1 ± 0.32	11.8 ± 1.2	3.6 ± 0.3	3.0 ± 0.2	4.5 ± 0.5
**MEF WT 48 h**	1.0 ± 0.2	5.8 ± 0.5	2.5 ± 0.3	0.9 ± 0.08	0.9 ± 0.1
